# Association between thrombus composition and stroke etiology in the MR CLEAN Registry biobank

**DOI:** 10.1007/s00234-023-03115-y

**Published:** 2023-01-25

**Authors:** Hajo M. Hund, Nikki Boodt, Daniel Hansen, Willem A. Haffmans, Geert J. Lycklama à Nijeholt, Jeannette Hofmeijer, Diederik W. J. Dippel, Aad van der Lugt, Adriaan C. G. M. van Es, Heleen M. M. van Beusekom, on behalf of the MR CLEAN Registry Investigators, Charles B. L. M. Majoie, Yvo B. W. E. M. Roos, Robert J. van Oostenbrugge, Wim H. van Zwam, Jelis Boiten, Jan Albert Vos, Ivo G. H. Jansen, Maxim J. H. L. Mulder, Robert- Jan B. Goldhoorn, Kars C. J. Compagne, Manon Kappelhof, Josje Brouwer, Sanne J. den Hartog, Wouter H. Hinsenveld, Bob Roozenbeek, Bart J. Emmer, Jonathan M. Coutinho, Wouter J. Schonewille, Marieke J. H. Wermer, Marianne A. A. van Walderveen, Julie Staals, Jasper M. Martens, Sebastiaan F. de Bruijn, Lukas C. van Dijk, H. Bart van der Worp, Rob H. Lo, Ewoud J. van Dijk, Hieronymus D. Boogaarts, J. de Vries, Paul L. M. de Kort, Julia van Tuijl, Jo P. Peluso, Puck Fransen, Jan S. P. van den Berg, Boudewijn A. A. M. van Hasselt, Leo A. M. Aerden, René J. Dallinga, Maarten Uyttenboogaart, Omid Eschgi, Reinoud P. H. Bokkers, Tobien H. C. M. L. Schreuder, Roel J. J. Heijboer, Koos Keizer, Lonneke S. F. Yo, Heleen M. den Hertog, Tomas Bulut, Paul J. A. M. Brouwers, Marieke E. S. Sprengers, Sjoerd F. M. Jenniskens, René van den Berg, Albert J. Yoo, Ludo F. M. Beenen, Alida A. Postma, Stefan D. Roosendaal, Bas F. W. van der Kallen, Ido R. van den Wijngaard, Joost Bot, Pieter-Jan van Doormaal, Anton Meijer, Elyas Ghariq, Marc P. van Proosdij, G. Menno Krietemeijer, Wouter Dinkelaar, Auke P. A. Appelman, Bas Hammer, Sjoert Pegge, Anouk van der Hoorn, Saman Vinke, H. Zwenneke Flach, Hester F. Lingsma, Naziha el Ghannouti, Martin Sterrenberg, Wilma Pellikaan, Rita Sprengers, Marjan Elfrink, Michelle Simons, Marjolein Vossers, Joke de Meris, Tamara Vermeulen, Annet Geerlings, Gina van Vemde, Tiny Simons, Gert Messchendorp, Nynke Nicolaij, Hester Bongenaar, Karin Bodde, Sandra Kleijn, Jasmijn Lodico, Hanneke Droste, Maureen Wollaert, Sabrina Verheesen, D. Jeurrissen, Erna Bos, Yvonne Drabbe, Michelle Sandiman, Nicoline Aaldering, Berber Zweedijk, Jocova Vervoort, Eva Ponjee, Sharon Romviel, Karin Kanselaar, Denn Barning, Esmee Venema, Vicky Chalos, Ralph R. Geuskens, Tim van Straaten, Saliha Ergezen, Roger R. M. Harmsma, Daan Muijres, Anouk de Jong, Olvert A. Berkhemer, Anna M. M. Boers, J. Huguet, P. F. C. Groot, Marieke A. Mens, Katinka R. van Kranendonk, Kilian M. Treurniet, Manon L. Tolhuisen, Heitor Alves, Annick J. Weterings, Eleonora L.F. Kirkels, Eva J. H. F. Voogd, Lieve M. Schupp, Sabine L. Collette, Adrien E. D. Groot, Natalie E. LeCouffe, Praneeta R. Konduri, Haryadi Prasetya, Nerea Arrarte-Terreros, Lucas A. Ramos

**Affiliations:** 1 Department of Cardiology, Erasmus MC University Medical Center, Room EE23.93, PO 2040, 3000CA Rotterdam, The Netherlands; 2 Department of Radiology, Haaglanden Medical Centrum, The Hague, The Netherlands; 3 Department of Radiology and Nuclear Medicine, Erasmus MC University Medical Center, Rotterdam, The Netherlands; 4 Department of Neurology, Erasmus MC University Medical Center, Rotterdam, The Netherlands; 5 Department of Neurology, Rijnstate Hospital, Arnhem, The Netherlands; 6 Department of Clinical Neurophysiology, University of Twente, Enschede, The Netherlands

**Keywords:** Ischemic stroke, Mechanical thrombectomy, Stent-retriever, Endovascular treatment, Thrombus, Microscopy

## Abstract

**Purpose:**

The composition of thrombi retrieved during endovascular thrombectomy (EVT) in acute ischemic stroke (AIS) due to large vessel occlusion (LVO) may differ depending on their origin. In this study, we investigated the association between thrombus composition and stroke etiology in a large population of patients from the Dutch MR CLEAN Registry treated with EVT in daily clinical practice.

**Methods:**

The thrombi of 332 patients with AIS were histologically analyzed for red blood cells (RBC), fibrin/platelets (F/P), and white blood cells (leukocytes) using a machine learning algorithm. Stroke etiology was assessed using the Trial of Org 10,172 in acute stroke treatment (TOAST) classification.

**Results:**

The thrombi of cardioembolic origin contained less RBC and more F/P than those of non-cardioembolic origin (25.8% vs 41.2% RBC [*p* = 0.003] and 67.1% vs 54.5% F/P [*p* = 0.004]). The likelihood of a non-cardioembolic source of stroke increased with increasing thrombus RBC content (*OR* 1.02; [95% *CI* 1.00–1.06] for each percent increase) and decreased with a higher F/P content (*OR* 1.02; [95% *CI* 1.00–1.06]). Thrombus composition in patients with a cardioembolic origin and undetermined origin was similar.

**Conclusion:**

Thrombus composition is significantly associated with stroke etiology, with an increase in RBC and a decrease in F/P raising the odds for a non-cardioembolic cause. No difference between composition of cardioembolic thrombi and of undetermined origin was seen. This emphasizes the need for more extensive monitoring for arrhythmias and/or extended cardiac analysis in case of an undetermined origin.

**Supplementary Information:**

The online version contains supplementary material available at 10.1007/s00234-023-03115-y.

## Introduction

With the emergence of endovascular thrombectomy (EVT) as part of standard treatment for acute ischemic stroke (AIS) caused by a large vessel occlusion (LVO), occluding thrombo-emboli have become available for histopathologic analysis. Insight in the relationship between thrombus composition and stroke etiology could be of value for secondary stroke prevention, as the appropriate preventive treatment depends on stroke etiology. Several causes of AIS have been identified, and the most prevalent being large artery atherosclerosis and cardiac embolism; both of which may lead to thrombo-embolic occlusions in the intracranial circulation. Stroke etiology currently remains unknown in approximately 30–50% of patients despite extensive clinical workup [1]. If a clear association exists between thrombus composition and stroke etiology, examination of extracted thrombi could be useful to guide therapeutic choices for secondary prevention of recurrent stroke [2].

Various previous studies have studied the relationship between thrombus composition and stroke etiology [3–21]. Most studies so far, however, did not find an association, possibly due to small sample sizes [3, 5, 6, 8, 11–15, 17]. While several studies did find an association between thrombus composition and etiology, they yielded contradictory results: some reported a higher amount of red blood cells (RBC) in thrombi associated with large artery atherosclerosis than in thrombi of cardiac or unknown origin [9, 10, 16, 18, 20, 21], while others reported the opposite with a higher amount of RBC in thrombi related to a cardiac source of embolization [7, 19, 22].

The aim of this study was to assess the relationship between thrombus composition and stroke etiology in a large population of AIS patients, treated with EVT for a large vessel occlusion of the anterior circulation, in daily clinical practice. In contrast to previous studies, we were able to compare clinical baseline characteristics of patients with and without thrombi available for analysis.

## Patients and methods

### Study population and sample selection

The MR CLEAN (Multicenter Randomized Clinical Trial of Endovascular Treatment for Acute Ischemic Stroke in the Netherlands) Registry was a prospective observational study of all patients who underwent EVT for AIS in the Netherlands (Appendix) [23]. Enrolment started March 2014, directly after the final inclusion in the MR CLEAN trial [24]. All patients undergoing EVT for AIS in the anterior or posterior circulation (defined as at least entry into the angiography suite and receiving arterial puncture), in one of the sixteen centers performing EVT in the Netherlands, were registered. The central medical ethics committee evaluated the study protocol and granted permission to carry out the study as a registry. For this histopathologic substudy, patients who met the following criteria were included: age 18 years and older, a proximal intracranial vessel occlusion in the anterior circulation as shown on CT angiography (CTA), availability of clinical and imaging data for the assessment of stroke etiology, and available thrombus for histological assessment. Data of patients treated until 15 June 2016 were collected and analyzed for this study.

### Thrombus analysis

After EVT, the thrombi were immediately stored in 4% buffered formaldehyde before embedding in paraffin. The thrombi were mostly retrieved by mechanical thrombectomy using a stent retriever, with only 14.8% using aspiration thrombectomy. For each paraffin block, 5-µm sections were cut at two depths, generally at a depth of 170 and 230 µm (Microm HM335 S, Microm International GmbH, Waldorf, Germany), as it has been shown that partial sectioning of the thrombus provides a good estimate of thrombus composition [25]. The two sections at these depths were collected on a single slide, stained by hematoxylin–eosin (HE), digitized at 20 × magnification (228 nm/pixel, Hamamatsu Nano-Zoomer, Hamamatsu Photonics K.K., Hamamatsu City, Japan), and images were stored as raw Hamamatsu.ndpi datafiles.

**Fig. 1:**
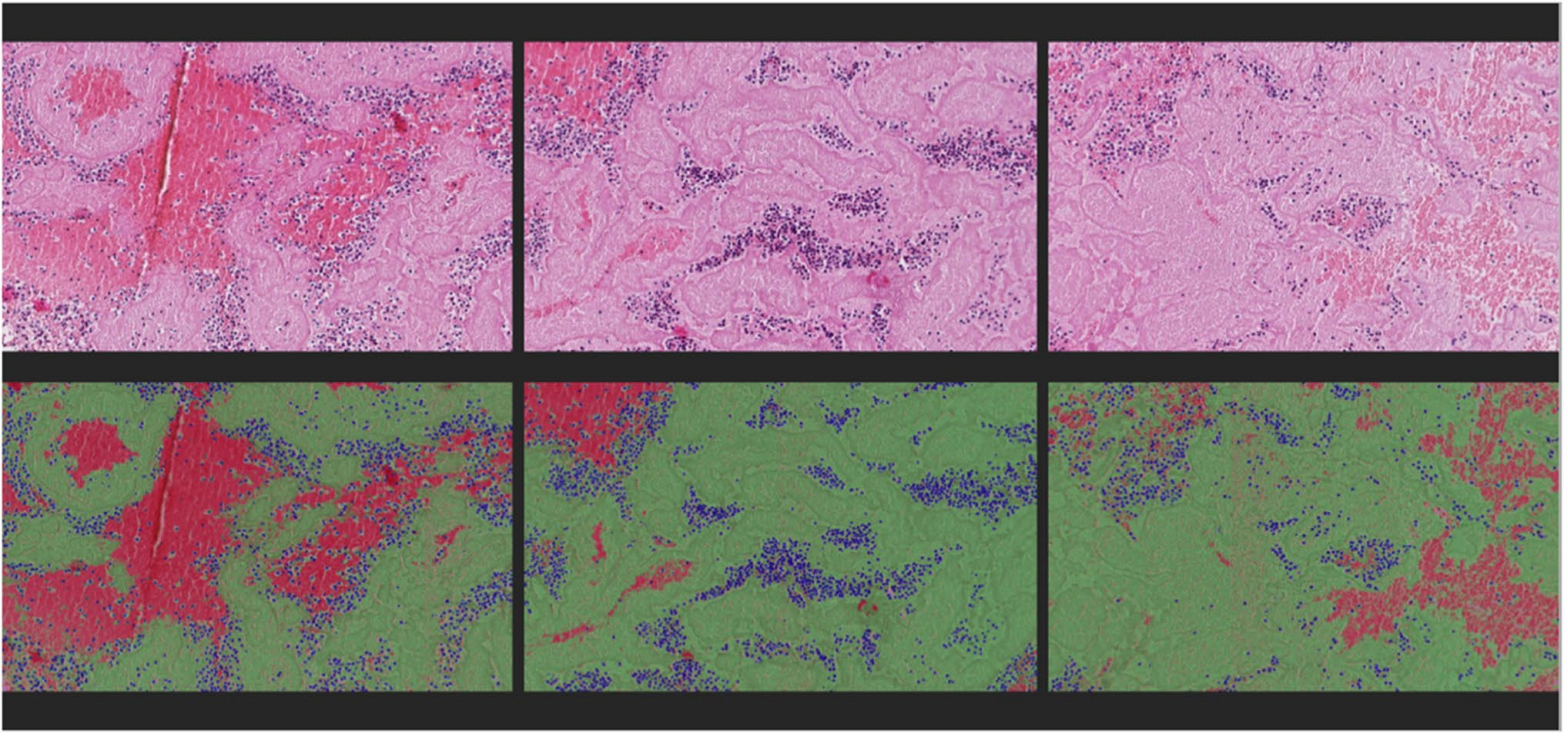
Examples of generated classification overlay images into RBC (red), F/P (green), and leukocytes (blue) to allow for visual inspection of the classification accuracy using Orbit. The top three images are original HE stained images; the bottom three images are the semi-automatically segmented images

An analysis of stained sections was performed using Orbit Image Analysis software (Orbit Image Analysis, Idorsia Ltd.) [26]. Orbit enables the analysis of native Hamamatsu.ndpi files and utilizes machine learning algorithms for image segmentation, classification, and quantification. First, a unified foreground/background segmentation model was trained to exclude background from further analysis. Using one unified classification model for all thrombi yielded less classification accuracy due to slight differences in staining between samples; therefore, separate classification models were created for each slide containing two sections. All models were trained to quantify percentages of red blood cells (RBC), fibrin and platelets (F/P), and white blood cells (leukocytes), as the main components of extracted EVT thrombi [3, 5–19, 22]. The weighted average of the two sections was considered representative for the whole thrombus [25]. Orbit was then used in conjunction with a custom script to enable batch analysis of all sections with individual classification models applied for each slide, generating both a Jason data file containing the quantification results per sample and a classification overlay image per sample (Fig. 1) to allow for visual inspection of the classification accuracy. Thrombus sections with large central defects in RBC rich sample regions, generally difficult to cut, were corrected by manually outlining and measuring these defects and adding the missing RBC surface area after comparison with the other section, and the original paraffin blocks (biomedical scientist (HH), validated by a pathobiologist (HB)).

### Assessment of stroke etiology

All patients underwent CTA or magnetic resonance angiography (MRA) of the cervical arteries before EVT. Furthermore, 12-lead ECG followed by ECG-monitoring for at least 24 h was performed. Additional etiologic workup was performed in accordance with local protocols. Stroke etiology was determined based on data provided in the discharge letters and imaging by two trained observers who were blinded for histological thrombus composition. The presumed cause of stroke was determined for each patient using the TOAST criteria as a guideline: large artery atherosclerosis (TOAST 1), cardioembolism (TOAST 2), stroke of other determined cause (TOAST 4), and stroke of undetermined cause (TOAST 5). In our cohort of patients who underwent EVT for AIS, there were no patients with small vessel disease as cause of stroke (TOAST 3). A patient was considered to have large artery atherosclerotic stroke, if there was > 50% atherosclerotic stenosis or atherosclerotic occlusion at the bifurcation of the carotid artery on the symptomatic side. Patients were considered to have undetermined stroke etiology, if more than one possible cause was identified; if no cause was identified despite complete workup, as described above; or if diagnostic workup was incomplete.

For statistical analysis, each patient was allocated to one of three predefined etiologic categories: (a) “non-cardioembolic” for both large artery atherosclerotic disease and other determined causes (TOAST 1 and 4), (b) “cardioembolic” (TOAST 2), or (c) “undetermined” (TOAST 5). For graphical representation of thrombus composition within groups, we further subdivided cardioembolic stroke into medium-risk or high-risk based on the evidence of the relative propensity for embolization, according to the original TOAST classification [27]. We also subdivided non-cardioembolic into large artery atherosclerosis, carotid artery dissection, and other determined cause. Stroke of undetermined etiology was subdivided in “ > 1 cause” (more than one possible cause was identified) and “cryptogenic” (no cause was identified despite complete workup, as described).

### Statistical analysis

Clinical characteristics and histological thrombus composition were described using standard statistics. Since thrombus composition did not follow a normal distribution, a Kruskal–Wallis test was performed first to assess differences in thrombus composition between all three etiologic groups (cardioembolic, non-cardioembolic, and undetermined). The association of histological components (percentages of RBC, F/P, and leukocytes) with stroke etiology (non-cardioembolic stroke, cardioembolic stroke, and stroke with undetermined etiology) was estimated with univariable and multivariable multinomial logistic regression and presented as (adjusted) odds ratios (*aOR*) with 95% confidence intervals (*CI*), with cardioembolic stroke as the reference category. We adjusted for potentially relevant differences in baseline characteristics by performing Kruskal–Wallis, *χ*^2^ test and Fisher-Freeman-Halton tests and including characteristics with a *p*-value of < 0.20 in the multivariable models (supplemental Table S1). For the regression analyses, single imputation was performed for missing values (supplemental Table S2). To assess the representativeness of the patients with an available thrombus to all patients who underwent EVT in clinical practice, we compared patient, clinical, and imaging characteristics of the included patients from the MRCLEAN Registry to those who were not included using ANOVA and Mann–Whitney *U* tests. Statistical analyses were performed using Stata (StataCorp. 2017. Stata Statistical Software: Release 15.1 SE. College Station, TX: StataCorp LLC) and SPSS (IBM Corp. Released 2020. IBM SPSS Statistics for Windows, Version 27.0. Armonk, NY: IBM Corp).

## Results

### Patient population

Thrombus samples of 332 patients from the MR CLEAN Registry were included for histological analysis in this study (Fig. S1). Baseline characteristics of these patients did not differ with those not included from the registry. Importantly, there were also no statistically significant differences in stroke etiology. For included patients, stroke etiology was categorized as cardioembolic in 114 patients (34.3%), non-cardioembolic in 58 patients (17.5%), and undetermined in 160 patients (48.2%), compared to 364 (30.5%), 199 (16.7%), and 631 (52.8%), respectively, in patients from the registry who were not included for histological analysis (Table 1).

**Table 1:** Baseline characteristics for patients from the MR CLEAN cohort with histological analysis as compared to those without. ANOVA was performed; *p*-values > 0.05 are printed bold

	Histology *n* = 332, (%)	No histology *n* = 1194, (%)	*p*-value
Age (median)	70	71	0.690^a^
Sex (male)	177 (53.3%)	632 (52.9%)	0.902
NIHSS baseline (median)	17	15	0.00^a^
IVT	251 (75.6%)	919 (77.2%)	0.551
Atrial fibrillation	92 (27.9%)	243 (20.7%)	**0.006**
Peripheral arterial disease	43 (13.3%)	95 (8.1%)	**0.004**
Previous stroke	66 (19.9%)	187 (15.8%)	0.072
Myocardial infarction	55 (16.8%)	178 (15.2%)	0.486
Antiplatelet use	105 (32.2%)	401 (34%)	0.555
Coumarin use	54 (16.5%)	140 (11.8%)	**0.025**
NOAC use	11 (3.4%)	26 (2.2%)	0.228
Occlusion segment based on CTA			
Intracranial ICA	12 (3.8%)	73 (6.5%)	0.072
ICA-T	90 (28.3%)	232 (20.5%)	**0.003**
M1	190 (59.7%)	652 (57.6%)	0.502
M2	25 (7.9%)	156 (13.8%)	**0.005**
Other: M3/anterior	1 (0.3%)	18 (1.6%)	0.077
Etiology (TOAST)			
Cardioembolic	114 (34.3%)	364 (30.5%)	0.181
Non-cardioembolic	58 (17.5%)	199 (16.7%)	0.729
Undetermined	160 (48.2%)	631 (52.8%)	0.133

^a^Mann-Whitney *U* test

### Differences in thrombus histology between etiologic groups

**Fig. 2:**
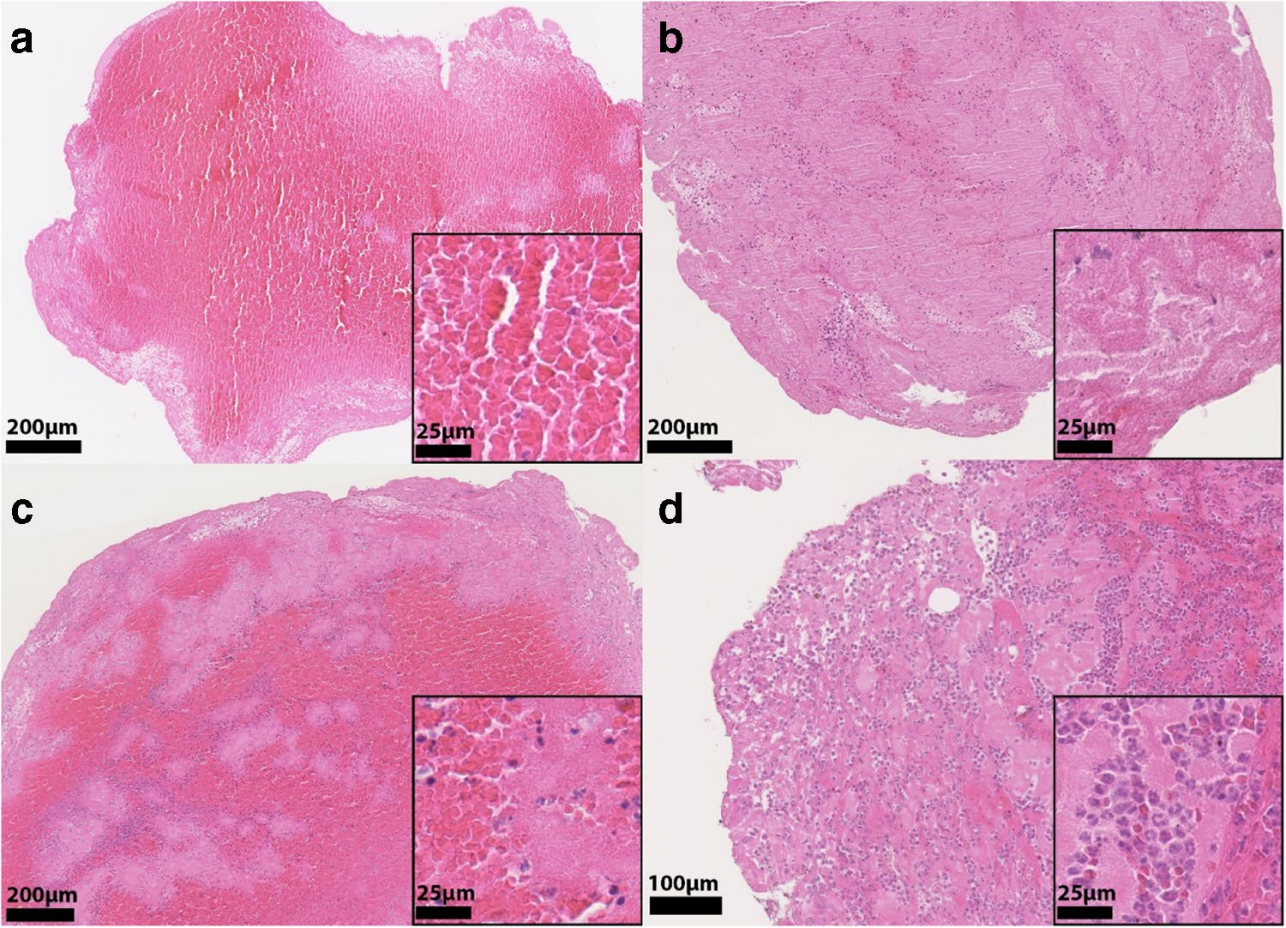
Examples of RBC rich (**A**), F/P rich (**B**), mixed (**C**), and leukocyte rich (**D**) thrombus areas. The inserted boxes represent magnifications of the underlying area of each example. HE-stain, bar = 200 µm (**A**–**C**), 100 µm (**D**), and 25 µm (inserts). RBC are red, F/P purple, and nuclei of leukocytes blue

**Fig. 3:**
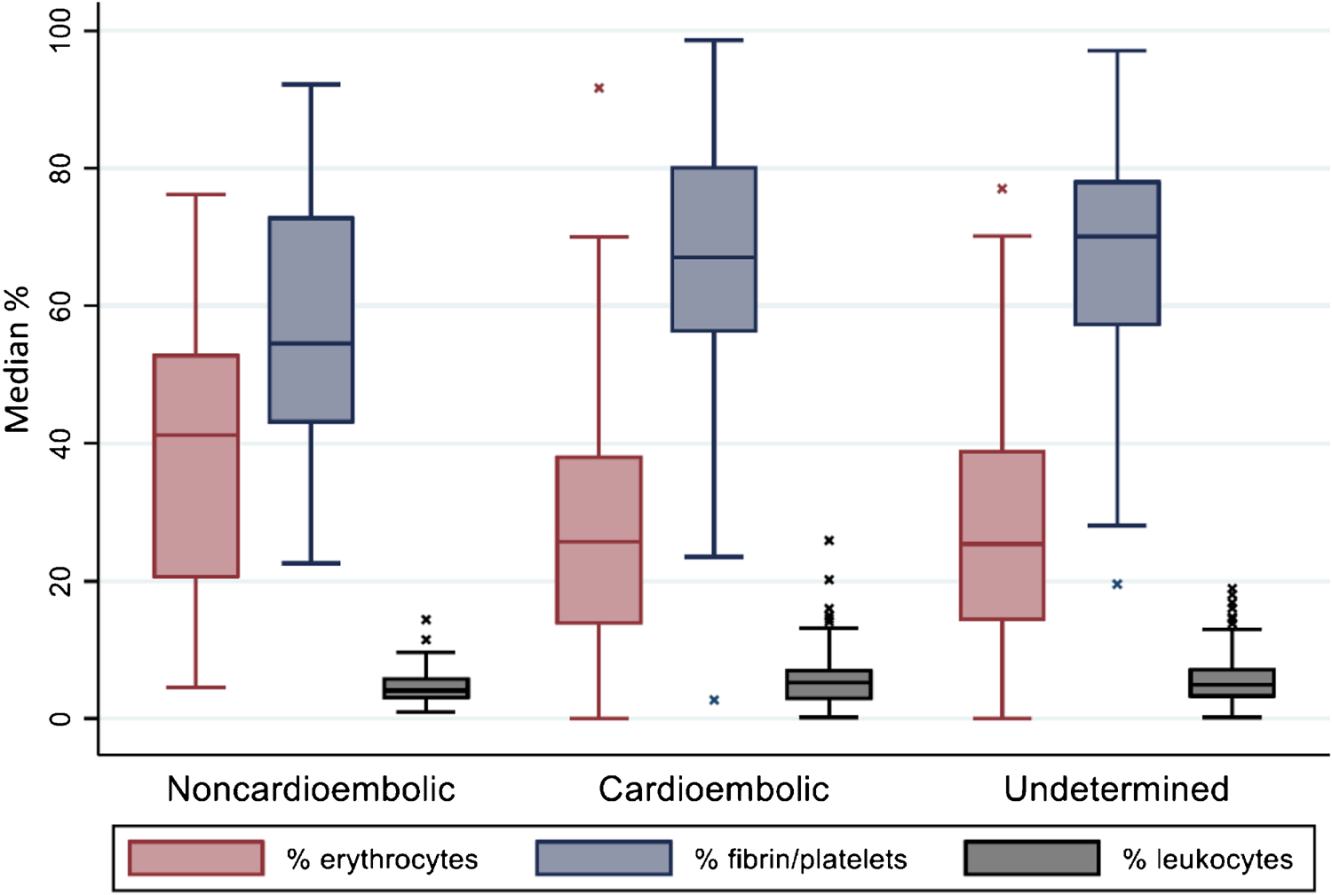
Bar-whisker plot of thrombus composition for the three etiologic groups. *P*-values are given based on Kruskal Wallis test. *x* = outliers > 1.5 times box height. Significant differences were found for both erythrocyte content (*p* < 0.001) and fibrin/platelet content (*p* = 0.002), but not for leukocytes (*p* = 0.240)

**Fig. 4:**
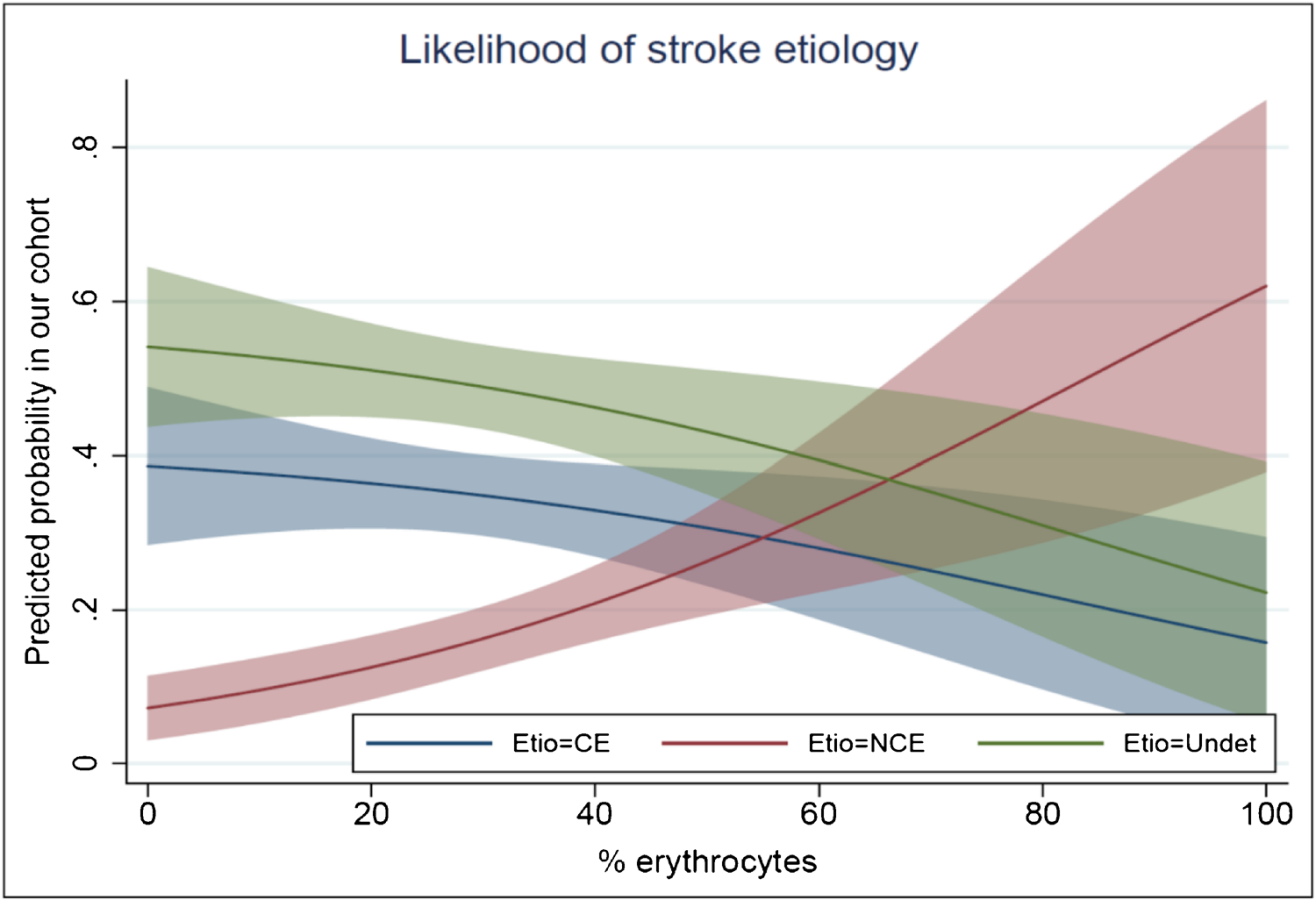
Likelihood of cardioembolic (CE), non-cardioembolic (NCE) and undetermined (Undet) etiology (Etio) based on erythrocyte content after univariable multinomial regression. For every 1% increase in RBC content, the *OR* for a non-cardioembolic stroke (as opposed to cardioembolic stroke) was 1.02

Histopathologic evaluation showed the thrombi typically contained areas being predominantly RBC, F/P, or leukocyte rich (Fig. 2). A large heterogeneity in overall composition was seen in our cohort (supplemental Fig. S2). For all thrombi, median RBC content was 27.1% (*IQR* 15.9–42.4), median F/P content was 67.0% (*IQR* 53.1–78.1) and median leukocyte content was 4.8% (*IQR* 3.0–7.1). RBC content in thrombi from patients with non-cardioembolic etiology (median 41.2%, *IQR* 20.5–53.0), was higher than in thrombi from patients with a cardiac etiology (median 25.8%, *IQR* 13.8–38.2) and thrombi from patients with an undetermined origin (median 25.4%, *IQR* 14.3–39.0). Inversely, the F/P content in thrombi from patients with non-cardioembolic etiology (median 54.5%, *IQR* 55.1–80.3) was lower than in thrombi from patients with cardioembolic etiology (median 67.1%, *IQR* 55.1–80.3) and thrombi with an undetermined origin (median 70.0%, *IQR* 57.1–78.2). Using a Kruskal–Wallis test for group differences, a significant difference was seen between the three etiologic groups for RBC content (*p* < 0.001) and F/P content (*p* = 0.002), but not for leukocyte content (*p* = 0.24) (Table 2 and Fig. 3). These differences were more pronounced for high-risk cardioembolic etiologies (supplemental Fig. S3). After correction for baseline differences in the regression analysis, RBC and F/P significantly differed between non-cardioembolic and cardioembolic strokes. Increased RBC (*OR*, 1.02 [95% *CI* 1.01–1.05]) and decreased F/P (*OR*, 0.98 [95% *CI* 0.95–0.99]) were associated with non-cardioembolic stroke, as opposed to cardioembolic stroke (Table 3). In other words, for every 1% increase in RBC content, the OR for a non-cardioembolic stroke (as opposed to cardioembolic stroke) was 1.02 (Fig. 4). Leukocyte content did not differ between non-cardioembolic and cardioembolic stroke. Furthermore, we did not observe any differences in thrombus composition between strokes with undetermined etiology and cardioembolic strokes in the univariable regression analyses (*OR* 1.00 [95% *CI* 0.99–1.01] for RBC, *OR* 1.00 [95% *CI* 0.99–1.01] for F/P and *OR* 1.00 [95% *CI* 0.94–1.07] for leukocytes) and multivariable regression analyses (Table 3).

**Table 2:** Thrombus composition stratified by stroke etiology

	Non-cardioembolic (*n* = 58)	Cardioembolic (*n* = 114)	Undetermined (*n* = 160)
Median (*IQR*) RBC %	41.2 (20.5–53.0)	25.8 (13.8–38.2)	25.4 (14.3–39.0)
Median (*IQR*) F/P %	54.5 (42.9–73.0)	67.1 (56.1–80.3)	70.0 (57.1–78.2)
Median (*IQR*) leukocytes %	4.1 (2.9–6.0)	5.2 (2.7–7.2)	5.0 (3.1–7.4)

**Table 3:** Univariable and multivariable multinomial logistic regression for the relationship of thrombus composition with stroke etiology

	Univariable model	Multivariable model*
Base outcome: cardioembolic	*OR* (95% *CI)*	*aOR* (95% *CI*)
Non-cardioembolic		
RBC	1.03 (1.01; 1.05)	1.02 (1.00; 1.04)
F/P	0.97 (0.95; 0.99)	0.98 (0.96; 1.00)
Leukocytes	0.91 (0.83; 1.01)	0.90 (0.81; 1.01)
Undetermined		
RBC	1.00 (0.99; 1.01)	1.00 (0.98; 1.01)
F/P	1.00 (0.99; 1.01)	1.00 (0.99; 1.02)
Leukocytes	1.00 (0.94; 1.07)	1.00 (0.92; 1.06)

All analyses were done with thrombus components as a continuous variable, expressed as % of the thrombus. Odds ratios for stroke etiology are shown per percentage increase of thrombus components, with 95% confidence intervals. Interpretation: for every 1% increase in RBCs, *OR* for a non-cardioembolic stroke (compared with cardioembolic stroke) is 1.03 (95% *CI* 1.01–1.05). Non-cardioembolic indicates TOAST 1 (large artery sclerosis) + TOAST 4 (other determined cause)

*RBC* red blood cells, *F/P* fibrin/platelets, and leukocytes white blood cells

^*^Adjusted for: age, sex, IV thrombolysis, coumarin/DOAC use, and thrombus location

## Discussion

We studied the association between histopathologic composition of mechanically extracted thrombi and stroke etiology in patients with AIS included in the Dutch MR CLEAN Registry. RBC and F/P contents of thrombi retrieved from patients with AIS differed significantly between strokes of cardioembolic and non-cardioembolic origin. The RBC content in thrombi from patients with non-cardioembolic etiology was higher than in thrombi from patients with a cardiac etiology and thrombi from patients with an undetermined origin. Inversely, the F/P content in thrombi from patients with non-cardioembolic etiology was lower than in thrombi from patients with cardioembolic etiology and thrombi with an undetermined origin. In addition, cardioembolic thrombi had a similar histopathologic composition to thrombi from strokes of undetermined origin.

Our findings of a significant correlation between stroke etiology and thrombus composition are in line with several previous studies [10, 16, 20, 21]. In our cohort, the wide confidence intervals and overlap in composition hamper reliable prediction of stroke etiology based on thrombus composition alone in individual cases. However, an increasingly high percentage of RBC in thrombi is associated with a higher likelihood of a non-cardioembolic etiology.

Like some previous studies [9, 10, 16, 21, 28], a similarity in thrombus composition was found for thrombi of cardioembolic origin and those of undetermined origin. Our results suggest that patients with an undetermined stroke origin and F/P rich thrombi have a higher likelihood of a cardiac source and may benefit from more extensive monitoring for arrhythmias and/or extended cardiac analysis.

Contradictory results in previous studies regarding the relationship between thrombus histology and etiology may in part be caused by random variation in relatively small samples, and a lack of consensus on histopathological processing and analysis [29]. Indeed, studies with larger sample sizes did find significantly higher fractions of RBC in ischemic stroke caused by large artery disease [10, 16, 20, 21], while smaller studies often failed to show such a correlation. Interestingly, all previous studies that found a higher RBC percentage in cardioembolic stroke were performed in an Asian population [9, 30, 31]. Lastly, many previous studies included thrombi from patients with posterior circulation stroke [3, 6, 8–10, 12, 13, 15, 18], while the determination of large artery atherosclerosis as a cause of AIS is based on stenosis grading of the anterior circulation.

Recently, studies have started to make use of machine learning software for image analysis [28, 30, 32], which implies training and validating a segmentation model first. Differences in accuracy of these segmentation models will affect quantification of thrombus components. In contrast to previous studies, we used a custom script in conjunction with Orbit image analysis software which enabled visual quality control in batch mode, enabling the possibility of verification of the segmented components RBC, F/P, and leukocytes by an experienced pathobiologist (HB). This diminished uncertainties normally inherent to the use of machine learning algorithms for segmentation purposes, ensuring high quality thrombus classification.

Our study has several limitations. Due to the ex vivo nature of thrombus analysis, all studies investigating the association between thrombus composition and stroke etiology theoretically suffer from a selection bias, since data from patients with thrombi resistant to thrombectomy as well as thrombi completely decomposed by IVT are not available for analysis. This study is the first to also have clinical data of all patients in the cohort without thrombus material available for analysis, enabling us to compare characteristics of included patients to those who were not included in the study. Indeed, this comparison was not possible in prior studies, including STRIP [20], through lack of sufficient clinical data in the LVO patient group as a whole. It is a feature that is available in, and a unique characteristic of, the MR CLEAN studies. We can therefore validate that no significant differences were found in stroke etiology between the two groups, and only minor differences in patient baseline characteristics were found. Therefore, we believe our results are valid for the general stroke population eligible for EVT. Using only HE staining makes a reliable differentiation between fibrin and platelets impossible; therefore, these components were combined into one category, F/P. Differentiation of platelets as a separate component and even adding more in-depth immunohistochemical analysis or even 3-D characterization of thrombi could certainly be of interest, as it has been recently shown that platelet content also differs between etiologic groups [32]. Also, since most patients received IVT prior to mechanical thrombectomy, it is possible that (partial) thrombolysis altered thrombus composition; we did, however, adjust for IVT in our regression analysis. Lastly, using the TOAST classification possibly underestimates the number of patients with large artery atherosclerosis as the cause for stroke, since it only values the degree of stenose at the carotid bifurcation. However, we did adhere to this classification, as it is the most used classification system in practice, enabling comparison of our results with previous studies.

## Conclusion

Thrombus composition is significantly associated with stroke etiology, with an increase in RBC and a decrease in F/P, raising the odds for a non-cardioembolic cause. Secondly, thrombus composition of cardioembolic and undetermined etiology are similar. These results suggest that patients with an undetermined origin and F/P-rich thrombi are more likely to have a cardiac cause and may benefit from more extensive monitoring for arrhythmias and/or extended cardiac analysis.

### Electronic supplementary material

Below is the link to the electronic supplementary material.

Supplementary file1 (DOCX 196 KB)

## Data Availability

The participants of this study did not give written consent for their data to be shared publicly, so due to the sensitive nature of the research, supporting data is not available.

## Appendix

MR CLEAN Registry Investigators — group authors

Executive committee

Diederik W. J. Dippel^1^, Aad van der Lugt^2^, Charles B. L. M. Majoie^3^, Yvo B. W. E. M. Roos^4^, Robert J. van Oostenbrugge^5^, Wim H. van Zwam^6^, Jelis Boiten^14^, Jan Albert Vos^8^

Study coordinators

Ivo G. H. Jansen^3^, Maxim J. H. L. Mulder^1,2^, Robert- Jan B. Goldhoorn^5,6^, Kars C. J. Compagne^2^, Manon Kappelhof^3^, Josje Brouwer^4^, Sanne J. den Hartog^1,2,40^, Wouter H. Hinsenveld^5,6^

Local principal investigators

Diederik W. J. Dippel^1^, Bob Roozenbeek^1^, Aad van der Lugt^2^, Adriaan C. G. M. van Es^2^, Charles B. L M. Majoie^3^, Yvo B. W. E. M. Roos^4^, Bart J. Emmer^3^, Jonathan M. Coutinho^4^, Wouter J. Schonewille^7^, Jan Albert Vos^8^, Marieke J. H. Wermer^9^, Marianne A. A. van Walderveen^10^, Julie Staals^5^, Robert J. van Oostenbrugge^5^, Wim H. van Zwam^6^, Jeannette Hofmeijer^11,41^, Jasper M. Martens^12^, Geert J. Lycklama à Nijeholt^13^, Jelis Boiten^14^, Sebastiaan F. de Bruijn^15^, Lukas C. van Dijk^16^, H. Bart van der Worp^17^, Rob H. Lo^18^, Ewoud J. van Dijk^19^, Hieronymus D. Boogaarts^20^, J. de Vries^22^, Paul L. M. de Kort^21^, Julia van Tuijl^21^, Jo P. Peluso^26^, Puck Fransen^22^, Jan S. P. van den Berg^22^, Boudewijn A. A. M. van Hasselt^23^, Leo A. M. Aerden^24^, René J. Dallinga^25^, Maarten Uyttenboogaart^28^, Omid Eschgi^29^, Reinoud P. H. Bokkers^29^, Tobien H. C. M. L. Schreuder^30^, Roel J. J. Heijboer^31^, Koos Keizer^32^, Lonneke S. F. Yo^33^, Heleen M. den Hertog^22^, Tomas Bulut^35^, Paul J. A. M. Brouwers^34^

Imaging assessment committee

Charles B. L. M. Majoie^3^ (chair), Wim H. van Zwam^6^, Aad van der Lugt^2^, Geert J. Lycklama à Nijeholt^13^, Marianne A. A. van Walderveen^10^, Marieke E. S. Sprengers^3^, Sjoerd F. M. Jenniskens^27^, René van den Berg^3^, Albert J. Yoo^38^, Ludo F. M. Beenen^3^, Alida A. Postma^6^, Stefan D. Roosendaal^3^, Bas F. W. van der Kallen^13^, Ido R. van den Wijngaard^13^, Adriaan C. G. M. van Es^2^, Bart J. Emmer^3^, Jasper M. Martens^12^, Lonneke S. F. Yo^33^, Jan Albert Vos^8^, Joost Bot^36^, Pieter-Jan van Doormaal^2^, Anton Meijer^27^, Elyas Ghariq^13^, Reinoud P. H. Bokkers^29^, Marc P. van Proosdij^37^, G. Menno Krietemeijer^33^, Jo P. Peluso^26^, Hieronymus D. Boogaarts^20^, Rob Lo^18^, Wouter Dinkelaar^2^, Auke P. A. Appelman^29^, Bas Hammer^16^, Sjoert Pegge^27^, Anouk van der Hoorn^29^, Saman Vinke^20^

Writing committee

Diederik W. J. Dippel^1^ (chair), Aad van der Lugt^2^, Charles B. L. M. Majoie^3^, Yvo B. W. E. M. Roos^4^, Robert J. van Oostenbrugge^5^, Wim H. van Zwam^6^, Geert J. Lycklama à Nijeholt^13^, Jelis Boiten^14^, Jan Albert Vos^8^, Wouter J. Schonewille^7^, Jeannette Hofmeijer^11,41^, Jasper M. Martens^12^, H. Bart van der Worp^17^, Rob H. Lo^18^

Adverse event committee

Robert J. van Oostenbrugge^5^ (chair), Jeannette Hofmeijer^11,41^, H. Zwenneke Flach^23^

Trial methodologist

Hester F. Lingsma^40^

Research nurses/local trial coordinators

Naziha el Ghannouti^1^, Martin Sterrenberg^1^, Wilma Pellikaan^7^, Rita Sprengers^4^, Marjan Elfrink^11^, Michelle Simons^11^, Marjolein Vossers^12^, Joke de Meris^14^, Tamara Vermeulen^14^, Annet Geerlings^19^, Gina van Vemde^22^, Tiny Simons^30^, Gert Messchendorp^28^, Nynke Nicolaij^28^, Hester Bongenaar^32^, Karin Bodde^24^, Sandra Kleijn^34^, Jasmijn Lodico^34^, Hanneke Droste^34^, Maureen Wollaert^5^, Sabrina Verheesen^5^, D. Jeurrissen^5^, Erna Bos^9^, Yvonne Drabbe^15^, Michelle Sandiman^15^, Nicoline Aaldering^11^, Berber Zweedijk^17^, Jocova Vervoort^21^, Eva Ponjee^22^, Sharon Romviel^19^, Karin Kanselaar^19^, Denn Barning^10^

PhD/Medical students

Esmee Venema^40^, Vicky Chalos1^40^, Ralph R. Geuskens^3^, Tim van Straaten^19^, Saliha Ergezen^1^, Roger R. M. Harmsma^1^, Daan Muijres^1^, Anouk de Jong^1^, Olvert A. Berkhemer^1,3,6^, Anna M. M. Boers^3,39^, J. Huguet^3^, P. F. C. Groot^3^, Marieke A. Mens^3^, Katinka R. van Kranendonk^3^, Kilian M. Treurniet^3^, Manon L. Tolhuisen^3,39^, Heitor Alves^3^, Annick J. Weterings^3^, Eleonora L.F. Kirkels^3^, Eva J. H. F. Voogd^11^, Lieve M. Schupp^3^, Sabine L. Collette^28,29^, Adrien E. D. Groot^4^, Natalie E. LeCouffe^4^, Praneeta R. Konduri^39^, Haryadi Prasetya^39^, Nerea Arrarte-Terreros^39^, Lucas A. Ramos^39^

^1^Department of Neurology, Erasmus MC University Medical Center, Rotterdam, The Netherlands

^2^Department of Radiology and Nuclear Medicine, Erasmus MC University Medical Center, Rotterdam, The Netherlands

^3^Department of Radiology and Nuclear Medicine, Amsterdam UMC, University of Amsterdam, Amsterdam, The Netherlands

^4^Department of Neurology, Amsterdam UMC, University of Amsterdam, Amsterdam, The Netherlands

^5^Department of Neurology, Maastricht University Medical Center and Cardiovascular Research Institute Maastricht (CARIM), Maastricht, The Netherlands

^6^Department of Radiology, Maastricht University Medical Center and Cardiovascular Research Institute Maastricht (CARIM), Maastricht, The Netherlands

^7^Department of Neurology, Sint Antonius Hospital, Nieuwegein, The Netherlands

^8^Department of Radiology, Sint Antonius Hospital, Nieuwegein, The Netherlands

^9^Department of Neurology, Leiden University Medical Center, Leiden, The Netherlands

^10^Department of Radiology, Leiden University Medical Center, Leiden, The Netherlands

^11^Department of Neurology, Rijnstate Hospital, Arnhem, The Netherlands

^12^Department of Radiology, Rijnstate Hospital, Arnhem, The Netherlands

^13^Department of Radiology, Haaglanden MC, The Hague, The Netherlands

^14^Department of Neurology, Haaglanden MC, The Hague, The Netherlands

^15^Department of Neurology, HAGA Hospital, The Hague, The Netherlands

^16^Department of Radiology, HAGA Hospital, The Hague, The Netherlands

^17^Department of Neurology, University Medical Center Utrecht, Utrecht, The Netherlands

^18^Department of Radiology, University Medical Center Utrecht, Utrecht, The Netherlands

^19^Department of Neurology, Radboud University Medical Center, Nijmegen, The Netherlands

^20^Department of Neurosurgery, Radboud University Medical Center, Nijmegen, The Netherlands

^21^Department of Neurology, Elisabeth-TweeSteden Ziekenhuis, Tilburg, The Netherlands

^22^Department of Neurology, Isala Klinieken, Zwolle, The Netherlands

^23^Department of Radiology, Isala Klinieken, Zwolle, The Netherlands

^24^Department of Neurology, Reinier de Graaf Gasthuis, Delft, The Netherlands

^25^Department of Radiology, Reinier de Graaf Gasthuis, Delft, The Netherlands

^26^Department of Radiology, Elisabeth-TweeSteden Ziekenhuis, Tilburg, The Netherlands

^27^Department of Radiology, Radboud University Medical Center, Nijmegen, The Netherlands

^28^Department of Neurology, University Medical Center Groningen, Groningen, The Netherlands

^29^Department of Radiology, University Medical Center Groningen, Groningen, The Netherlands

^30^Department of Neurology, Atrium Medical Center, Heerlen, The Netherlands

^31^Department of Radiology, Atrium Medical Center, Heerlen, The Netherlands

32Department of Neurology, Catharina Hospital, Eindhoven, The Netherlands

^33^Department of Radiology, Catharina Hospital, Eindhoven, The Netherlands

^34^Department of Neurology, Medisch Spectrum Twente, Enschede, The Netherlands

^35^Department of Radiology, Medisch Spectrum Twente, Enschede, The Netherlands

^36^Department of Radiology, Amsterdam UMC, Vrije Universiteit van Amsterdam, Amsterdam, The Netherlands

^37^Department of Radiology, Noordwest Ziekenhuisgroep, Alkmaar, The Netherlands

^38^Department of Radiology, Texas Stroke Institute, TX, USA

^39^Department of Biomedical Engineering & Physics, Amsterdam UMC, University of Amsterdam, Amsterdam, The Netherlands

^40^Department of Public Health, Erasmus MC University Medical Center, Rotterdam, The Netherlands

^41^Department of Clinical Neurophysiology, University of Twente, Enschede, The Netherlands
