# Supplementary material for: Association between thrombus composition and stroke etiology in the MR CLEAN Registry biobank
Source: Neuroradiology. 2023 Jan 25;65(5):933–43. doi: 10.1007/s00234-023-03115-y (PMC10105654; doi:10.1007/s00234-023-03115-y)
Supplement: Supplementary file 1 — Supplementary file1 (DOCX 196 KB) [file 234_2023_3115_MOESM1_ESM.docx]

**SUPPLEMENTAL TABLES AND FIGURES**

**Figure S1.** Inclusion flow-chart MR-CLEAN registry thrombus substudy.


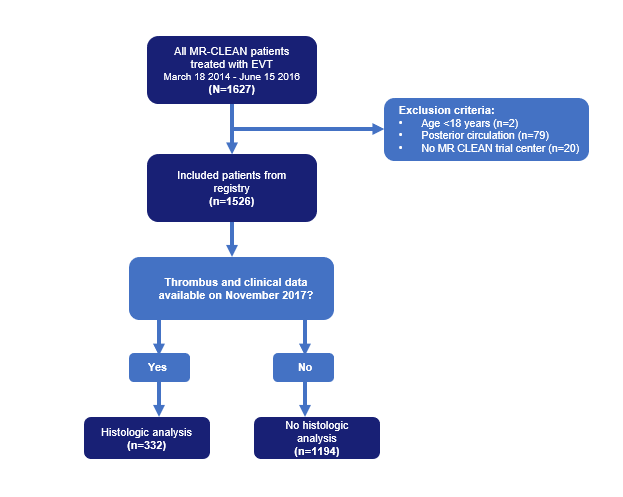


**Table S1.** Baseline characteristics per TOAST group (n= 332). Statistical analysis was performed, and relevant parameters with P-values ≤ 0,20 were included as co-variables in the linear regression analysis (in bold). ^a^ Kruskall-Wallis test. ^b^ χ^2^ test. ^c^ Fisher-Freeman-Halton test.

|  | Cardioembolic (n=114) | Noncardioembolic (n=58) | Undetermined (n=160) | P-value |
| --- | --- | --- | --- | --- |
| Age median | 74 | 67 | 69 | **0.000^a^** |
| Sex male (%) | 54 (47.4) | 38 (65.5) | 85 (53.1) | **0.084^b^** |
| Baseline NIHSS median | 17 | 18 | 17 | 0.438^a^ |
| IVT (%) | 66 (57.9) | 48 (82.8) | 137 (85.6) | **0.000^b^** |
| Peripheral arterial disease (%) | 18 (16.2) | 9 (16.1) | 16 (10.3) | 0.294^b^ |
| Previous stroke (%) | 24 (21.1) | 11 (19.3) | 31 (19.4) | 1.000^c^ |
| Myocardial infarction (%) | 15 (13.5) | 11 (19.0) | 29 (18.4) | 0.503^b^ |
| Antiplatelet use (%) | 31 (27.9) | 21 (36.8) | 53 (33.5) | 0.450^b^ |
| Coumarin use (%) | 40 (35.7) | 1 (1.8) | 13 (8.2) | **0.000^b^** |
| NOAC use (%) | 8 (7.2) | 1 (1.8) | 2 (1.3) | **0.033^c^** |
| Occlusion segment based on CTA |  |  |  |  |
| Intracranial ICA (%) | 1 (0.9) | 5 (8.6) | 6 (3.9) | **0.039^c^** |
| ICA-T (%) | 26 (24.3) | 21 (36.2) | 43 (28.1) | 0.276^b^ |
| M1 (%) | 69 (64.5) | 31 (53.4) | 90 (58.8) | 0.368^b^ |
| M2 (%) | 10 (9.3) | 1 (1.7) | 14 (9.2) | 0.150^c^ |
| Other: M3/anterior (%) | 1 (0.9) | 0 (0.0) | 0 (0.0) | 0.519^c^ |

**Table S2.** Variables used for single imputation and their frequency of missing values.

| **Variable** | **Frequency of missing values, n total (%)** | **Frequency of missing values, n histology (%)** |
| --- | --- | --- |
| Age | 0/1526 | 0/1526 |
| Sex | 0/1526 | 0/1526 |
| Time from onset to groin | 0/1526 | 0/1526 |
| IVT | 3/1526 (0) | 0/1526 |
| Coumarin use | 11/1526 (1) | 4/332 (0) |
| DOAC use | 26/1526 (2) | 7/332 (2) |
| Occlusion segment | 77/1526 (5) | 14/332 (4) |

**Figure S2.** Thrombus composition distribution graph of all 332 thrombi analyzed shows large heterogeneity of the thrombus components RBC, F/P and leukocytes within the analyzed cohort.

**Figure S3.** Bar-whisker plots of thrombus composition showing the percentage RBC (red), F/P (blue) and leukocytes (grey) for etiologic groups after subdividing into large artery atherosclerosis (LAA), medium risk cardioembolic (CE Mediumrisk), high risk cardioembolic (CE Highrisk), other (other), carotid artery dissection (CAD), more than one cause identified (> 1cause) and unknown etiology after standard clinical follow-up (cryptogenic). Top: Median percentage RBC (red), F/P (blue Median). Bottom: percentage leukocytes (grey). P-values given based on Mann-Whitney U tests. °=outliers > 1.5 times box height.


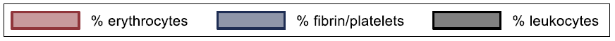

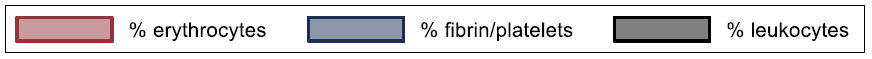

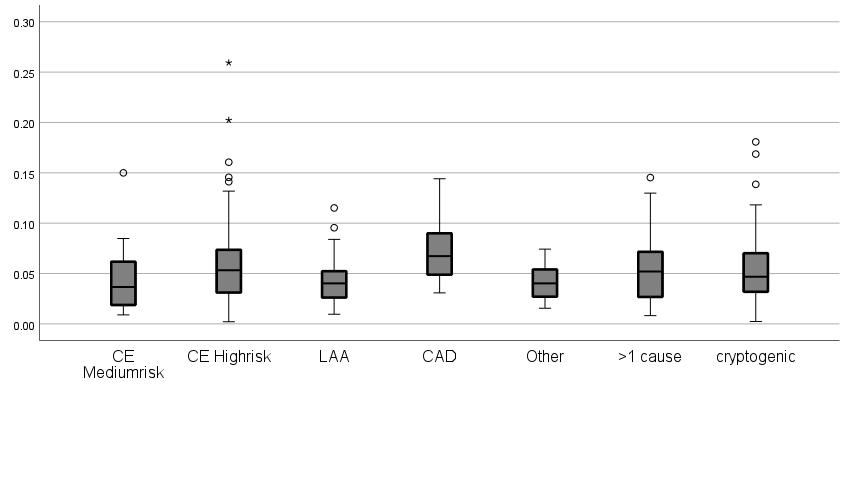

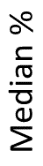

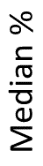

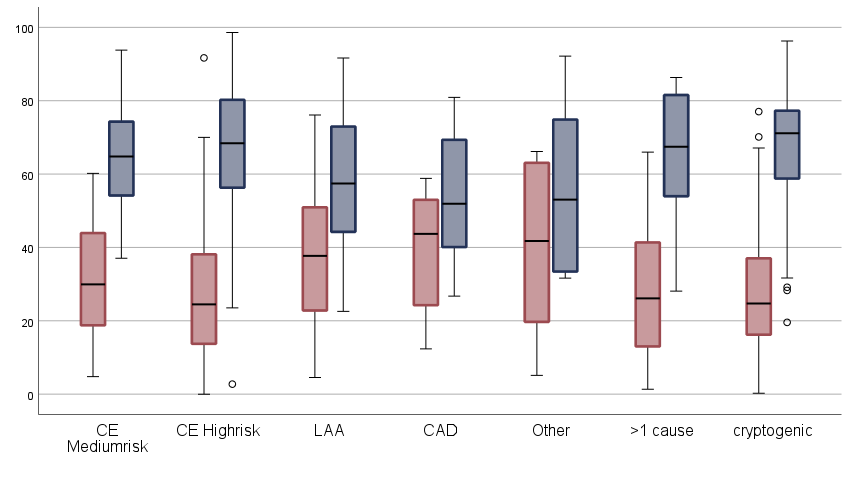


**CUSTOM SCRIPT FOR BATCH ANALYSIS:**

Importantly, our script only enhances the batch processing capabilities of Orbit image analysis software, it does not change the way the software analyses the thrombus sections. The source code of Orbit analysis is publicly available on GitHub (https://github.com/mstritt/orbit-image-analysis). Our script to speed up batch analysis, as well as a brief ‘read-me’ on how to use it, will be provided upon request.
